# Phlegmasia cerulea dolens in a patient with partial inferior vena cava interruption treated with mechanical thrombectomy

**DOI:** 10.1016/j.jvscit.2025.101912

**Published:** 2025-07-07

**Authors:** Alexander S. Misono, Avinash Mesipam, Tust Techasith

**Affiliations:** Division of Interventional Radiology, Department of Radiology, Hoag Hospital Irvine, Irvine, CA

**Keywords:** Deep venous thrombosis, Venous thromboembolism, Partial inferior vena cava interruption, Mechanical thrombectomy, Phlegmasia cerula dolens

## Abstract

Phlegmasia cerulea dolens is a rare condition associated with deep vein thrombosis (DVT). Inferior vena cava clips are even more rarely seen devices historically placed in the context of DVT. In this case, a 70-year-old woman with a distant history of DVT presented with acute onset left calf pain, swelling, and discoloration. The patient was discovered to have decreased arterial pulses on physical examination, but no evidence of acute arterial occlusion. Upon further workup, an occlusive left pelvic DVT as well as partial inferior vena cava interruption via a Moretz clip was discovered. The patient was treated with mechanical thrombectomy and iliocaval reconstruction. Patients with complex and altered venous anatomy may be prone to complications of DVT. They remain candidates for endovascular thrombectomy and stenting. In fact, these techniques may represent the most effective and efficient treatments in these circumstances.

Inferior vena cava (IVC) filtration has been a part of deep vein thrombosis (DVT) management for decades. Originally pioneered in the 1950s and becoming more common in the 1960s, partial interruption of the IVC was a surgical technique designed to prevent embolism in patients with known DVT.^1^, ^2^, ^3^, ^4^, ^5^ Indications for placement commonly included anticoagulation failure, anticoagulation contraindication, and prophylaxis.^6^^,^^7^ Clip types included the Adams-DeWeese clip, Moretz clip, and Miles clip, each with its own distinct characteristics.^8^^,^^9^ In some patients, these clips have been implicated in chronic venous disease as well as acute DVT. Iliocaval reconstruction and thrombolysis have been described in patients with partial IVC interruption.^10^, ^11^, ^12^ We present a unique case of mechanical thrombectomy in the treatment of phlegmasia cerulea dolens in a patient with partial IVC interruption. The patient consented to the publication of these case details and images.

## Case report

A 70-year-old woman presented to the emergency department with acute and progressive severe left leg pain, swelling, tightness, discoloration, and paresthesia for 1 day. Significant past medical history included a distant history of DVT, type 2 diabetes, hyperlipidemia, and hypothyroidism.

The patient reported struggling with ambulation owing to her acute symptoms. On examination, she was noted to have a dusky-appearing, swollen, and taut left lower extremity. Her left pedal pulses were not palpable, whereas her right pedal pulses were palpable. Initial vital signs and laboratory studies in the emergency department were notable for an elevated white blood cell count (13,600) and elevated lactate level (5.2 mmol/L).

**Fig 1:**
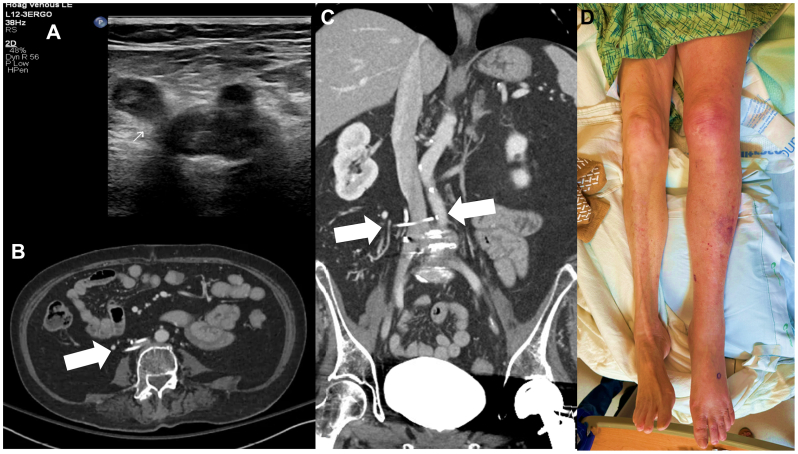
The patient presented with an acutely discolored and swollen left lower extremity with progressive paresthesia. **(A)** Venous ultrasound examination demonstrates occlusive thrombus throughout the left lower extremity, including at the saphenofemoral junction, as shown here. **(B)** Computed tomography (CT) venogram axial image demonstrates left lower extremity thrombosis (not shown) and also reveals an Moretz inferior vena cava (IVC) clip encircling the lower IVC (*white arrow*). **(C)** CT venogram coronal reformat shows again the IVC clip in place (*white arrows*). **(D)** Picture of patient legs showing markedly swollen and taut left lower extremity with purpuric discoloration. Bedside evaluation revealed nonpalpable left pedal pulses, whereas right pedal pulses were easily palpable. Left pedal pulses are audible only on Doppler ultrasound examination.

Arterial duplex ultrasound examination was performed initially, which revealed no arterial occlusion. Subsequently, venous ultrasound examination was performed, showing an occlusive DVT in the left lower extremity involving the common femoral vein (Fig 1, *A*). Contrast-enhanced computed tomography (CT) scan of the abdomen/pelvis with venogram protocol was subsequently performed, demonstrating the presence of a Moretz IVC interruption clip encompassing the lower IVC (Fig 1, *B* and *C*). The CT scan also demonstrated acute-on-chronic DVT in the left external and common iliac veins. On further questioning, the patient recalled undergoing an IVC surgery in the 1960s owing to a diagnosis of DVT and an unclear contraindication to anticoagulation. Given the diagnosis of extensive DVT, decreased left arterial pedal pulses, lactic acidemia, and progressive paresthesia, a diagnosis of phlegmasia cerulea dolens was established.

**Fig 2:**
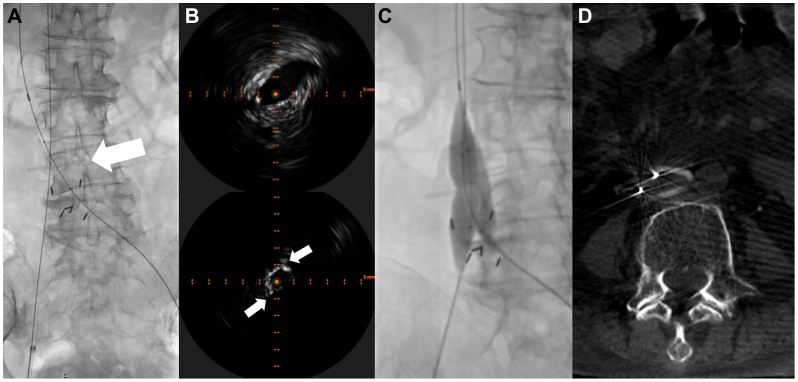
The patient was taken for endovascular venography and intervention. **(A)** In a prone position, bilateral popliteal venous access was obtained and the left lower extremity venous thrombosis as well as the inferior vena cava (IVC) clip was crossed. The Moretz clip is faintly visible (*white arrow*). **(B)** IVUS images demonstrate normal-appearing IVC (top) and echogenic linear signal in keeping with the Moretz clip (bottom; *white arrows*). **(C)** Balloon venoplasty is performed with two, 14-mm angioplasty balloons in a kissing fashion. **(D)** A cone beam computed tomography (CT) scan was performed, demonstrating deformation and expansion of the clip into more of an oval configuration.

The patient was initiated on a heparin infusion, and a treatment plan was devised. The patient was brought urgently to the angiography suite for diagnostic venography and definitive endovascular intervention. The patient was placed in a prone position and the bilateral popliteal veins were accessed with ultrasound guidance and Seldinger techniques. Then, 9F vascular sheaths were placed in each popliteal vein. Conventional catheters and wires were used to cross the iliofemoral veins. Subsequently, the Moretz clip was crossed with ease, establishing an 0.035” wire access across the DVT as well as partial IVC interruption (Fig 2, *A*). Intravascular ultrasound examination was performed, demonstrating transition from a normal-appearing IVC to a characteristic linear echogenic appearance of the Moretz clip with luminal restriction (Fig 2, *B*). Then, kissing balloon venoplasty was performed with 14-mm angioplasty balloons to deform and expand the Moretz clip intentionally ahead of the planned thrombectomy and reconstruction (Fig 2, *C* and *D*).

**Fig 3:**
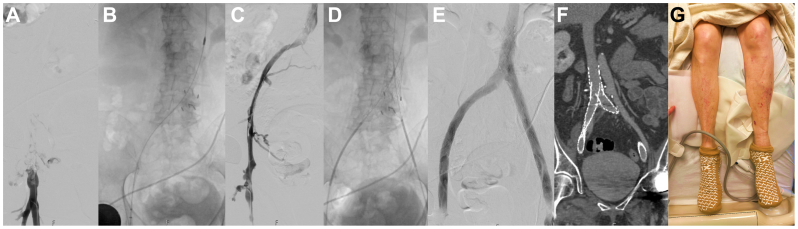
Attention was then turned to endovascular mechanical thrombectomy for deep vein thrombosis (DVT). **(A)** A venogram demonstrated occlusive thrombus in the left common femoral, external iliac, and common iliac veins. **(B)** Mechanical thrombectomy was pursued with a ClotTriever Bold catheter. **(C)** After thrombectomy, there was re-establishment of flow in the iliac veins, albeit with chronic-appearing long-segment stenosis and webs. **(D)** Iliocaval reconstruction was pursued. In the inferior vena cava (IVC), a 20 × 6 cm Abre stent was placed. Kissing 14 mm × 15 cm (left iliac) and 14 mm × 12 cm (right iliac) Abre stents were placed. **(E)** Completion venogram showed widely patent iliocaval reconstruction. **(F)** Postoperative computed tomography (CT) venogram demonstrated patent iliocaval reconstruction, with a single slice focusing on the right iliac reconstruction. **(G)** Patient legs at 24 hours after intervention showing significant improvement in swelling, size, and purpura of the left lower extremity, with near normal appearance.

Thereafter, attention was turned to the left lower extremity DVT. An initial venogram demonstrated occlusive thrombosis of the left common femoral, external iliac, and common iliac veins (Fig 3, *A*). After dilation, a 13F ClotTriever Sheath (Inari Medical, Irvine, CA) was placed into the left popliteal vein access. A ClotTriever Bold catheter (Inari Medical) was then placed over the wire and mechanical thrombectomy was performed by pulling the device retrograde from the common iliac vein to the common femoral vein (Fig 3, *B*). No mechanical or physical interactions or issues were seen related to the Moretz clip during the mechanical thrombectomy maneuver. After the thrombectomy, a venogram demonstrated recanalization of the treated segment, although there was compression of the left common iliac vein by the overlying right common iliac artery and long-segment stenosis of the left common-to-external iliac vein and iliocaval confluence, as well as chronic venous scarring/webbing (Fig 3, *C*). This finding was further confirmed on IVUS examination.

After thrombectomy, iliocaval reconstruction was performed with the intention to buttress both the clipped IVC but also treat the aforementioned chronic stenoses at the iliocaval confluence and the left iliac vein. A 20 mm × 6 cm Abre stent (Medtronic, Minneapolis, MN) was first advanced into the IVC and placed across the partial IVC interruption. This was postdilated with an 18-mm balloon. Subsequently, kissing 14-mm iliac stents were placed to complete the reconstruction (Fig 3, *D*). Completion venogram and subsequent CT venogram demonstrate a widely patent reconstruction (Fig 3, *E* and *F*).

After completion of the intervention, the patient immediately reported improvement in symptoms. Her left lower extremity pedal pulses normalized within 4 hours after the intervention. Her lactic acidemia and leukocytosis normalized by morning laboratory testing on postoperative day 1. The patient remained in the hospital for 1 week owing to comorbid factors. The postoperative course was complicated by anemia, requiring active management of anticoagulation. Ultimately, the patient was discharged on a direct oral anticoagulant with a plan for life-long anticoagulation. Follow-up clinical evaluation demonstrated patency of the iliocaval reconstruction and complete resolution of the presenting symptoms.

## Discussion

This case demonstrates a unique combination of distant technology and modern endovascular techniques. Phlegmasia cerulea dolens is the most severe complication of DVT resulting from complete venous outflow obstruction leading to tissue ischemia. It is important to distinguish this entity from phlegmasia alba dolens, in which venous obstruction is partial. The ability to perform endovascular thrombectomy rapidly is critical in patients with phlegmasia cerulea dolens. With increasing comfort, the management of such patients even in the context of uncommonly seen variations such as partial IVC interruption is feasible.

In this case, it is likely that the severe presentation of phlegmasia cerulea dolens was the result of a combination of several factors, including acute-onset DVT, chronic venous fibrosis, stenosis of the ipsilateral iliac veins, and superimposed partial IVC interruption. Although original studies on partial IVC interruption demonstrated a significant effect on hemodynamics, it is likely that, over a period of decades, an undesirable effect on hemodynamics may be inevitable.^5^ Furthermore, the presence of acute-on-chronic DVT alone can precipitate this dangerous physiology.

Endovascular reconstruction in patients with partial IVC interruption has been described, most commonly in the context of chronic venous disease or insufficiency.^10^, ^11^, ^12^ Venous thrombolysis has also been incorporated into the management of patients with superimposed acute DVT.^10^ However, to our knowledge, there has not been a report of mechanical thrombectomy in a patient with partial IVC interruption, although such techniques have been seen in other IVC pathologies, such as oversewing. With this case report, the safety and efficacy of mechanical thrombectomy technology appears to be validated further in cases of IVC pathology.

In emergent situations where revascularization of the DVT is of clinical importance, mechanical thrombectomy may be pursued, even in the context of complex venous pathology inclusive of partial IVC interruption.

## Conclusions

Phlegmasia cerulea dolens and partial IVC interruption are both rare entities, even more so when combined with acute-on-chronic DVT. Mechanical thrombectomy solutions may provide safe and effective recanalization in such cases.

## Funding

None

## Disclosures

A.S.M. is a consultant for AIDoc, Argon Medical, MediView, Medtronic, Merit Medical, SinglePass, Sirtex, Stryker, Terumo, and TriSalus Medical. A.S.M. is a speaker for AIDoc, Argon Medical, Medtronic, Merit Medical, SinglePass, Sirtex, Stryker, TriSalus Medical, and Vasorum. A.S.M. has received research funding from Inari Medical.
